# Supplementary material for: Implicit adaptation’s effect on sensorimotor and motor confidence
Source: bioRxiv. 2025 Dec 17:2025.12.15.694412. Preprint. [Version 1] doi: 10.64898/2025.12.15.694412 (PMC12724612; doi:10.64898/2025.12.15.694412)
Supplement: Supplement 1 [file NIHPP2025.12.15.694412v1-supplement-1.pdf]

bioRxiv preprint doi: https://doi.org/10.64898/2025.12.15.694412; this version posted December 17, 2025. The copyright holder for this preprint (which was not certified by peer review) is the author/funder, who has granted bioRxiv a license to display the preprint in perpetuity. It is made available under aCC-BY 4.0 International license.

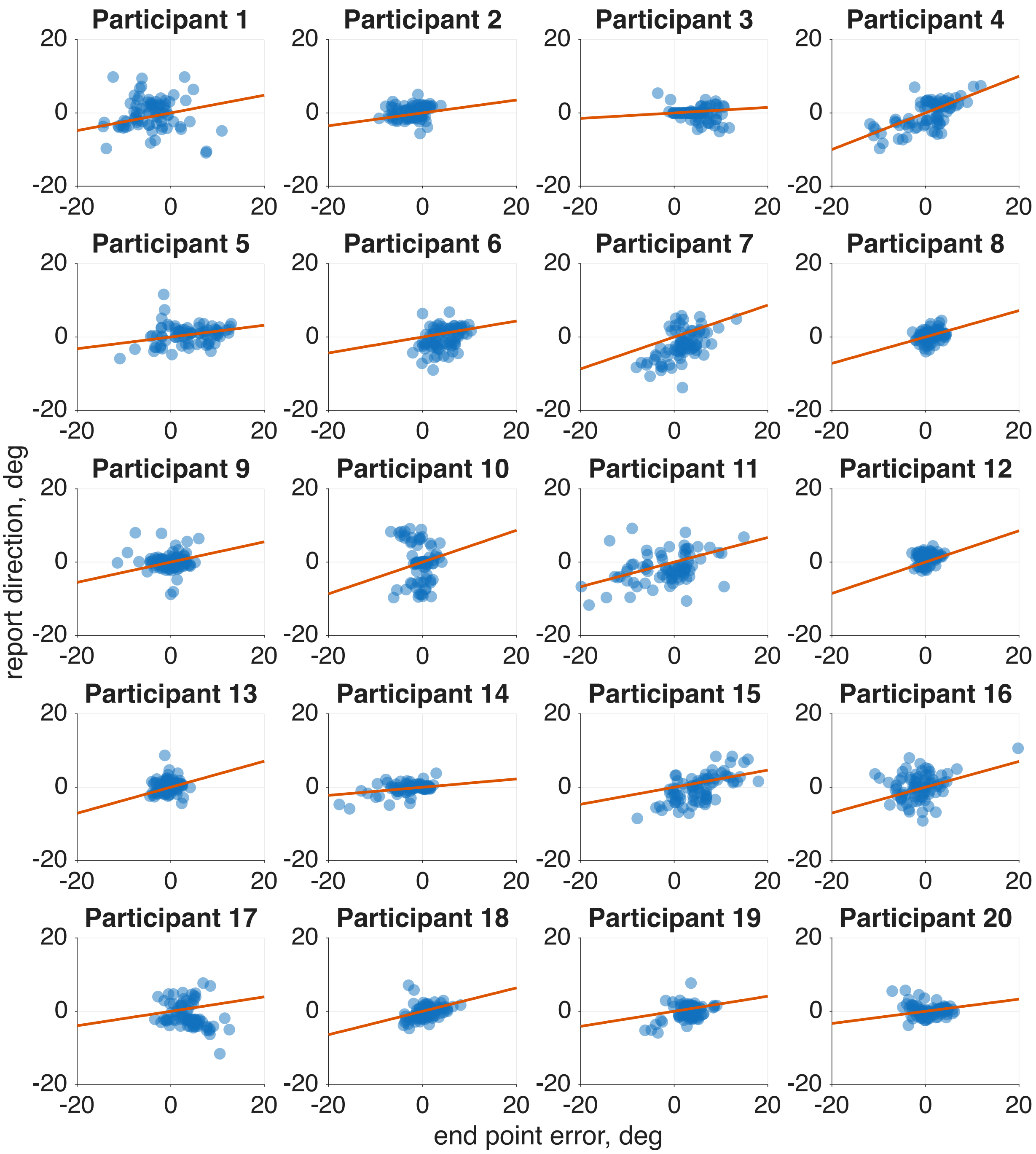

Supplement 1: Motor awareness model fit.
For all twenty participants the end point error and reported reach direction were plotted and overlaid with a line following the slope of the reliabilities of the parameters fit by the model. We can see that our model captures trends within the data.

bioRxiv preprint doi: https://doi.org/10.64898/2025.12.15.694412; this version posted December 17, 2025. The copyright holder for this preprint (which was not certified by peer review) is the author/funder, who has granted bioRxiv a license to display the preprint in perpetuity. It is made available under aCC-BY 4.0 International license.

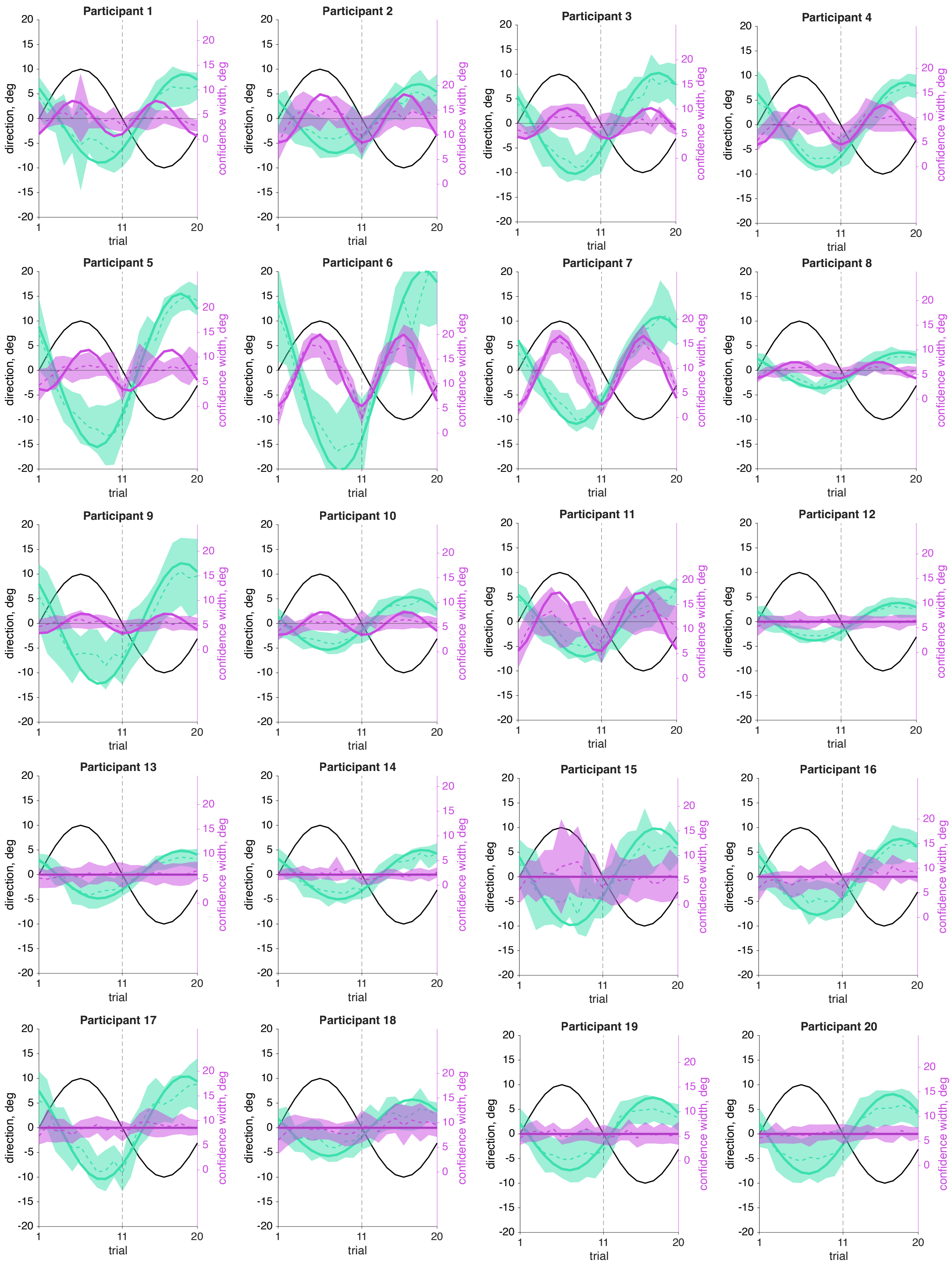

bioRxiv preprint doi: https://doi.org/10.64898/2025.12.15.694412; this version posted December 17, 2025. The copyright holder for this preprint (which was not certified by peer review) is the author/funder, who has granted bioRxiv a license to display the preprint in perpetuity. It is made available under aCC-BY 4.0 International license.

Supplement 2: Sensorimotor confidence averaged time courses

An averaged cycle (dashed line with shaded CI) and an overlayed sine wave (solid line) using the phase and amplitude components from a Fourier analysis at the expected frequency of the response is shown here for all twenty participants. The sine wave has phase and amplitude from a Fourier analysis at the frequency of 24 cycles per session when that amplitude was significantly different from zero (Participants 1-11). For the other participants the mean is plotted (i.e., 0 amplitude). All participants showed a significant 12 cycles per session frequency for reach direction (teal).

bioRxiv preprint doi: https://doi.org/10.64898/2025.12.15.694412; this version posted December 17, 2025. The copyright holder for this preprint (which was not certified by peer review) is the author/funder, who has granted bioRxiv a license to display the preprint in perpetuity. It is made available under aCC-BY 4.0 International license.

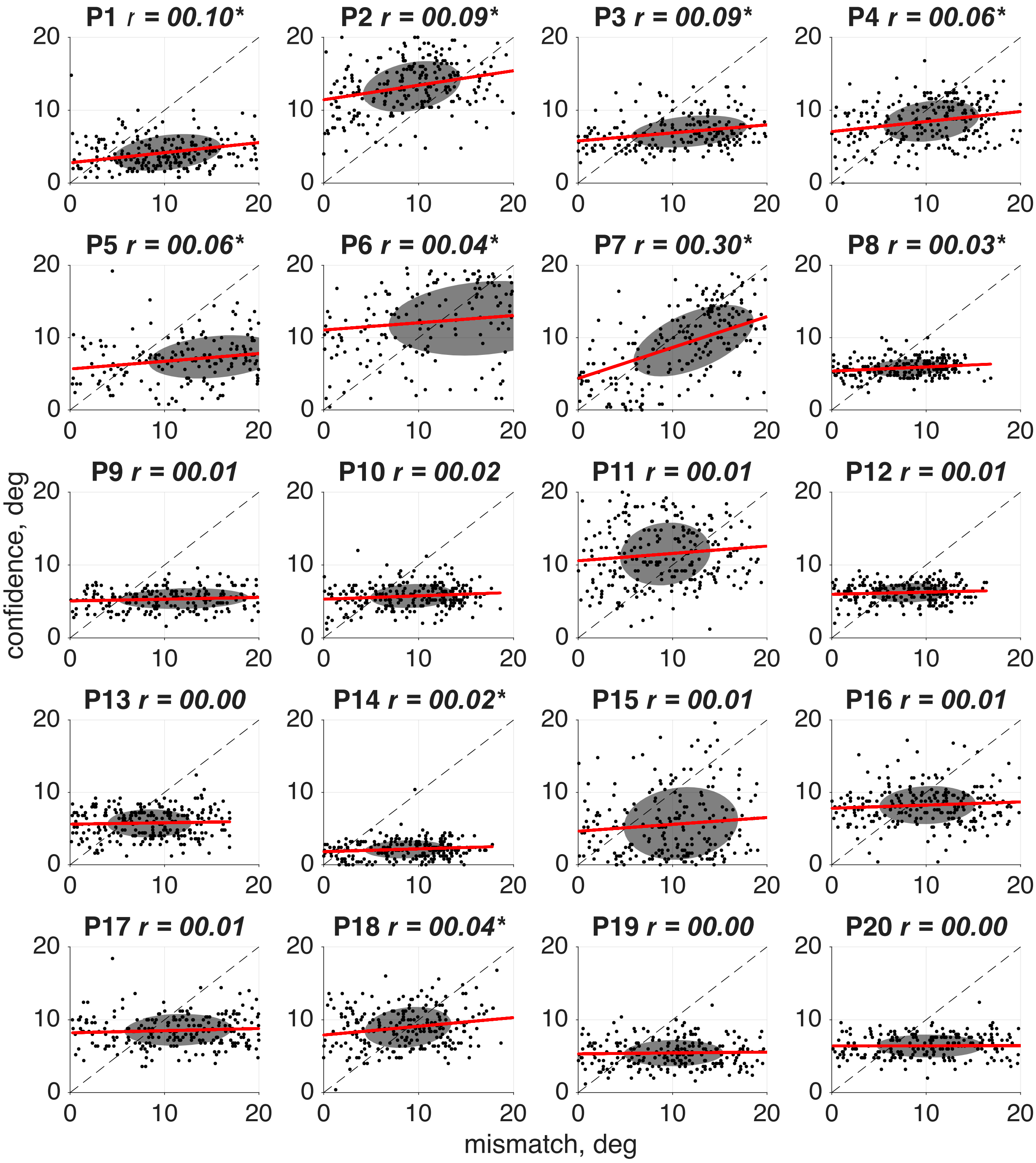

Supplement 3: Correlation between sensorimotor confidence and the mismatch between hand and feedback position.

Correlation between the confidence report on a given trial and the distance (in degrees) between the visual feedback location and hand position for the same four sample participants. Since the reach lag is on average 1.89 trials past true anti-phase, the point at which the mismatch is the smallest occurs when the error clamp is slightly off center from the target.

bioRxiv preprint doi: https://doi.org/10.64898/2025.12.15.694412; this version posted December 17, 2025. The copyright holder for this preprint (which was not certified by peer review) is the author/funder, who has granted bioRxiv a license to display the preprint in perpetuity. It is made available under aCC-BY 4.0 International license.

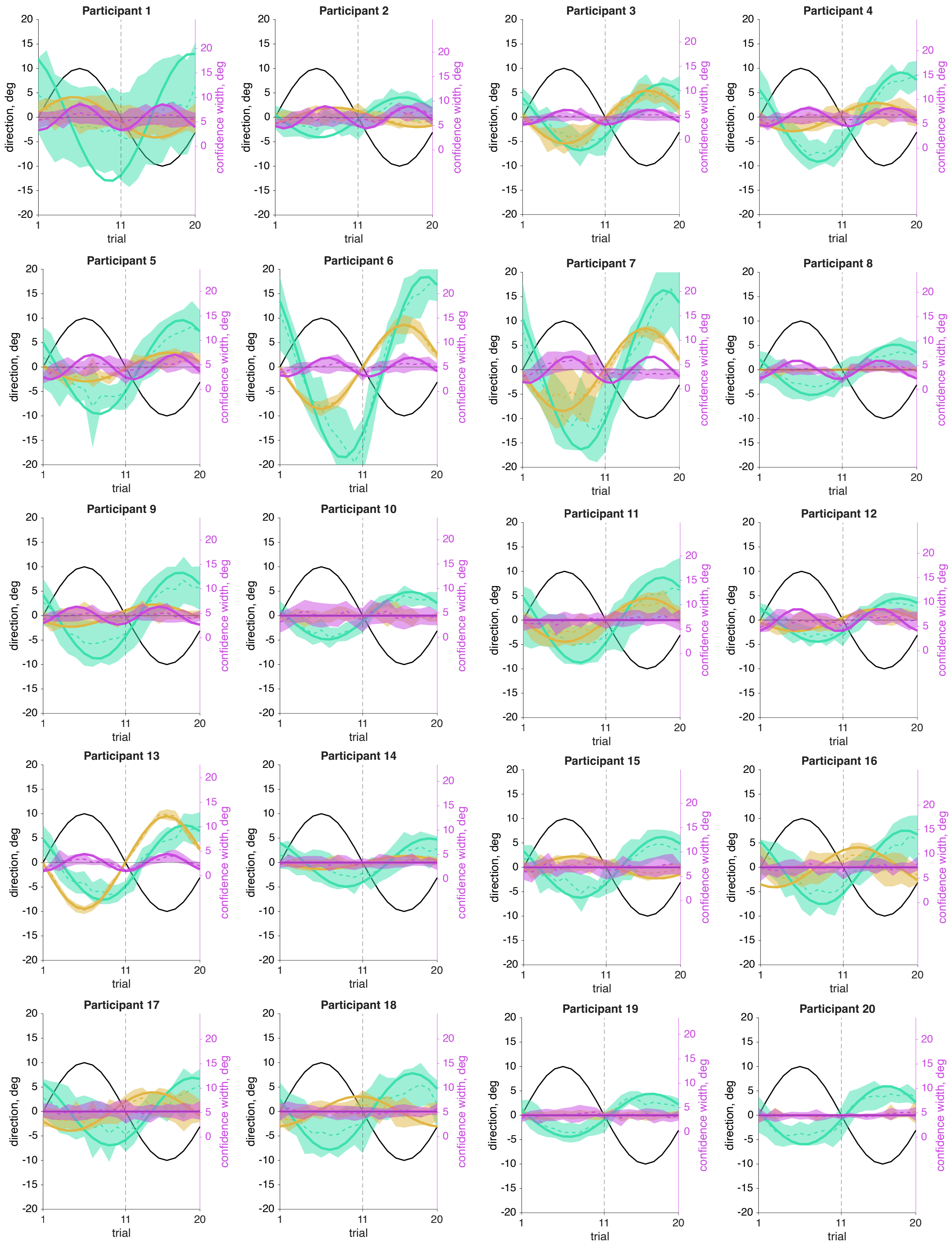

bioRxiv preprint doi: https://doi.org/10.64898/2025.12.15.694412; this version posted December 17, 2025. The copyright holder for this preprint (which was not certified by peer review) is the author/funder, who has granted bioRxiv a license to display the preprint in perpetuity. It is made available under aCC-BY 4.0 International license.

Supplement 4: Motor-awareness confidence averaged time courses

An averaged cycle (dashed line with shaded CI) and an overlayed sine wave (solid line) using the phase and amplitude components from a Fourier analysis at the expected frequency of the response is shown here for the same four sample participants. Participants 1-9 & 12-13 had a significant 24 cycles per session frequency in confidence (magenta), while the other participants did not show this significant frequency component (and so the sine is plotted at zero amplitude). All participants showed a significant 12 cycles per session frequency for reach direction (teal). Participants 19 & 20 do not show a significant 12 cycles per session frequency in the reported hand position (tan), but the other participants all show notably different trial lags of the report relative to the error clamp.

bioRxiv preprint doi: https://doi.org/10.64898/2025.12.15.694412; this version posted December 17, 2025. The copyright holder for this preprint (which was not certified by peer review) is the author/funder, who has granted bioRxiv a license to display the preprint in perpetuity. It is made available under aCC-BY 4.0 International license.

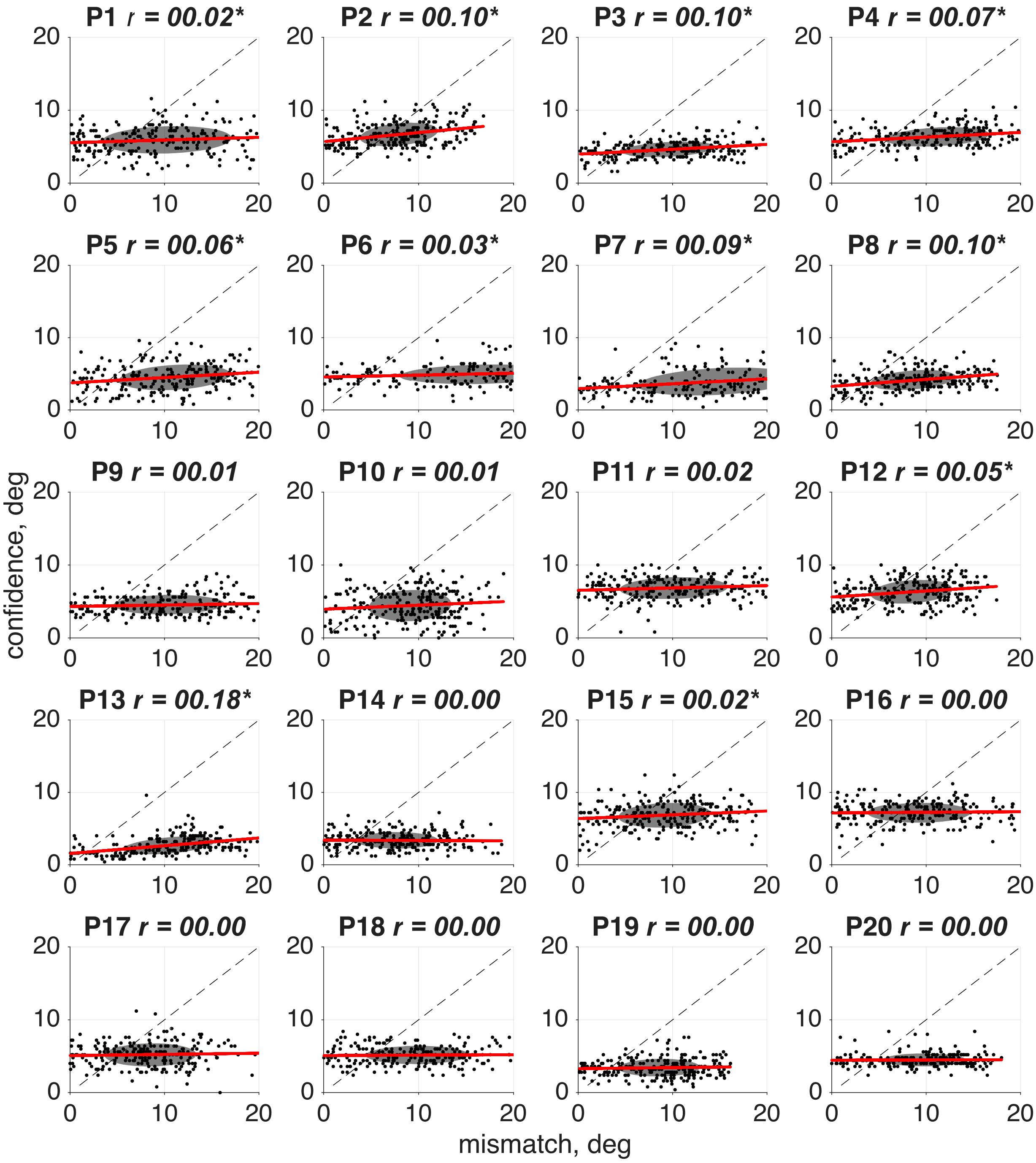

Supplement 5: Correlation between motor-awareness confidence and the mismatch between hand and feedback position.

Correlation between the confidence report on a given trial and the distance (in degrees) between the visual feedback location and hand position for the same four sample participants. Since the reach lag is on average 1.84 trials past true anti-phase, the point at which the mismatch is the smallest is not centered at the target position, 0º.
